# Factors associated with the appropriate use of preoperatory hospital stays: historical cohort study

**DOI:** 10.1186/1472-6963-7-187

**Published:** 2007-11-19

**Authors:** Sonia Tamames, Alberto Perez Rubio, Javier Castrodeza Sanz, Maria Belen Canton Alvarez, Francisco J Luquero, Sara Santos Sanz, Placido Lopez Encinar, Maria Paz de la Torre Pardo, Juan Manuel Gil Gonzalez

**Affiliations:** 1 Quality Unit. University Clinical Hospital. Valladolid, Spain; 2 Medical Management. University Clinical Hospital. Valladolid, Spain

## Abstract

**Background:**

To ensure the highest efficiency, health services should be provided with the least possible complexity. The aim of this study is to quantify the degree of appropriateness in preoperatory hospital stays and to analyse those factors associated with a greater inappropriate use.

**Methods:**

Historical cohort study. The histories of 440 hospitalised patients who underwent at least one surgical procedure were analysed. Data collection was carried out by doctors not involved in the services studied, following the Appropriateness Evaluation Protocol. A bivariate and multivariate analysis of the factors associated with the appropriateness of preoperatory stays was carried out.

**Results:**

The mean number of days of preoperatory stay was 5.5 (SD 5.11), of which a mean number of 2.5 days were considered to be inappropriate (SD 4.11). The overall rate of inappropriateness was 45.2% (CI 95% 43.3–47.1). The multivariate analysis showed a positive association of the inappropriateness of the preoperatory stay with weekend days, programmed admission, hospital stays longer than 7 days, medical records incorrectly or incompletely documented and the age groups of 45–65 and the >65 with respect to the <45 age group. Sex and an incorrect or incomplete nursing register did not show such an association.

**Conclusion:**

The inappropriate use of hospital stay during preoperatory care affects almost half the period and there are some risk determinants that could act as indicators at admission. In addition, the efficiency of care provision was found to vary greatly from the point of view of its appropriateness.

## Background

Increased healthcare expenditure in developed countries is influenced by several complex factors, among which can be numbered the population's own expectations concerning their health and their demographic structure [1]. The ability to guarantee the sustainability of Europe's healthcare systems, without detriment to accessibility or efficiency, is an ever growing preoccupation. Some of the proposed strategies for keeping down expenditure on healthcare include the provision of services at an 'as simple as possible assistance level', thus optimising the use of available resources [2]. In this sense, a review of the appropriate use of hospital care is especially relevant, as it allows inappropriate stays and admissions to be identified and minimised [3].

Of the different evaluation methods [4], the one that is most widely used and validated in our setting is the Appropriateness Evaluation Protocol (AEP)  [5]. These methods, linked to the programmes guaranteeing quality of care, are aimed at reducing unnecessary care, from the clinical point of view, but without questioning the indication of medical or nursing care provided. They only consider the assistance level and the moment at which the healthcare is given, as a particular characteristic of the AEP. This evaluation protocol defines as unnecessary or inappropriate stay that which could have been carried out at a lower assistance level, in less time or with better programming [6].

Since the acceptance of the AEP in Spain, many works have been published that have investigated and described the degree of inappropriate use, both medical and surgical. The published papers vary greatly in their methodology and the type of patient considered  [7]. In addition, as far as we know, there have been no specific analyses of concrete periods within the hospitalisation episode. The aim of this study is to quantify the degree of appropriateness in preoperatory hospital stays, these being especially susceptible to inappropriateness as the care provision during this period can more feasibly be given at assistance levels of a lower complexity. We also propose to analyse which factors are associated with a greater inappropriate use.

## Methods

This is a historical cohort study, which is part of a wider study of the same characteristics to evaluate the appropriateness of hospital stays. The study was carried out in the University Clinical Hospital of Valladolid, a teaching and public hospital serving a total population of 261,105 people. The hospital has 777 beds, of which 360 are surgical. Twelve thousand two hundred and fifty four patients were admitted to the 'Surgical Area' in 2006. The hospital belongs to the healthcare system of 'Castilla y León', a region situated on the northern plain of Spain. The study design is non-interventional and retrospective, and was conducted in accordance with the Spanish and European Union regulations regarding health research and data protection and approved by the hospital administration team. It also complies with the Declaration of Helsinki of 1968, as amended in 2000.

The inclusion criteria were: patients already selected by means of random sampling from the admission database for a one-year period to participate in the wider study previously mentioned, admitted and discharged by any of the twelve surgical services included in the analysis, who underwent at least one surgical procedure during their stay and whose preoperatory stay was equal to, or longer than, one day. We consider 'preoperatory stay' to be the time between the date of admission and the date of the first procedure, without including either of these days. The exclusion criteria were: obstetrics patients and those who were admitted to services not included in the analysis.

From the initial sample of 1630 patients for studying the evaluation of the appropriateness of hospital stays, a total of 440 patients were found to be adequate for the present analysis. This sample size guarantees a precision in estimating parameters of at least 5%. The main variable was appropriateness and the following were included as possible independent variables: age and sex of the patient, the type of admission (programmed or urgent), the duration of the preoperatory period (one week or more), the existence of correctly documented medical records (at least every 24 hours and at least consigning 'without changes' for the whole hospitalization episode) and nursing records (at least every 8 hours and at least consigning 'without changes' for the whole hospitalization episode), whether each day was a working day (Monday to Friday) or not, and the service to which the patient was admitted. Each preoperatory day after admittance was taken as the unit of analysis. The day when the first procedure was carried out and the subsequent days were not taken into account.

The measuring instrument used was the Appropriateness Evaluation Protocol [6] validated for use in Spain [5], which includes a list of criteria to justify the appropriateness of hospitalization. Should the criteria not be fulfilled, then the evaluated stay is considered to be inappropriate. The AEP allows a series of causes of inappropriateness, which have not been considered in this analysis, to be identified. The evaluation was carried out by means of a review of each patient's case history performed by 6 doctors not belonging to the services studied, following an initial period of training.

Data collection and analysis was carried out using MS Access^® ^and the statistical package SPSS^® ^version 13.0. First, descriptive analyses of the admittances and stays included in the study were obtained. A bivariate analysis was then carried out to evaluate the association between the inappropriateness of the stay and the variables described above by means of Relative Risk (RR) with a Confidence Interval at 95% (CI95%) estimated by the Katz method. Statistical significance was assessed using Pearson's χ^2 ^statistic. Finally, a multivariate analysis was carried out using logistic regression by the Enter method.

## Results

From the initial 1630 patients, 904 met the criteria of having been admitted and discharged by any of the twelve surgical services included in the analysis, 464 of them were excluded because no procedure was performed on them or because their preoperatory stay was less than one day. Finally, 440 patients were found to meet inclusion and exclusion criteria. The mean age of the said patients was 62.6 (Standard Deviation, SD 16.17), of whom 61.1% were male (CI95% 55.3–67.0). These patients had spent a total of 2,584 days in their preoperatory stay. The mean number of days of preoperatory stay was 5.5 (SD 5.11), the median number of days was 3 (range 1–35). An average of 2.5 days were considered to be inappropriate (SD 4.11), with a median value of 1 day (range 0–33). The distribution of the subjects according to the factors analysed in the study, as well as the surgical services included, are shown in table 1.

**Table 1:** Characteristics of hospital admissions.

**PATIENTS**^†^		**n**	**%**	95CI-LL*	95CI-UL*
**Age**	< 45 years	62	14.1	10.7	17.5
	45–65 years	158	35.9	28.4	43.4
	> 65 years	220	50.0	43.4	56.6
**Sex**	Male	171	38.9	31.6	46.2
	Female	269	61.1	55.3	67.0
**Admission**	Programmed	278	63.2	57.5	68.9
	Urgent	162	36.8	29.4	44.2
**Hospitalization length**	< 7 days	324	73.6	68.8	78.4
	≥ 7 days	116	26.4	18.3	34.4
**Correctly documented medical records**	Yes	121	27.5	19.5	35.5
	No	319	72.5	67.6	77.4
**Correctly documented nursing records**	Yes	421	95.7	93.7	97.6
	No	19	4.3	2.3	6.3
**Service**	Angiology and Vascular Surgery	60	13.6	10.3	17.0
	Heart Surgery	24	5.5	3.2	7.7
	General Surgery 'Service A'	67	15.2	11.8	18.7
	General Surgery 'Service B'	28	6.4	4.0	8.8
	Laparoscopic Surgery	19	4.3	2.3	6.3
	Thoracic Surgery	34	7.7	5.1	10.3
	Gynaecology	17	3.9	1.9	5.8
	Neurosurgery	19	4.3	2.3	6.3
	Ophthalmology	12	2.7	1.1	4.4
	Otorhinolaryngology	22	5.0	2.85	7.2
	Traumatology	84	19.1	10.7	27.5
	Urology	54	12.3	9.1	15.5
**STAYS**^‡^					
**Working day**	Yes	1971	76.3	74.4	78.2
	No	613	23.7	20.4	27.1

*95CI: 95% confidence interval; LL: lower limit; UL: upper limit. ^† ^distribution of patients by personal and hospital admissions' characteristics. ^‡ ^distribution of the total number of stays generated by patients included.

The overall rate of inappropriateness was found to be 45.2% (CI95% 43.3–47.1). In the bivariate analysis, the inappropriateness of the stays was associated with: 1) weekends as opposed to working days, 2) programmed admissions as opposed to urgent admissions, 3) the greater the patient's age, 4) the preoperative stay over one week and 5) incorrect or incomplete medical records. No statistically significant differences were found as to inappropriateness with respect to sex or nursing records (table 2).

**Table 2:** Percentage of inappropriate stays and associated factors: bivariate and multivariate analysis.

			**Rate of inappropriateness**			**Bivariate analysis**				**Multivariate analysis**			
			
		**n**	**%**	95CI-LL*	95CI-UL*	RR*	95CI-LL*	95CI-UL*	**p**	ROR*	95CI-LL*	95CI-UL*	**p**
**Age**	< 45 years^†^	257	29.2	23.6	34.7								
	45–65 years	968	50.3	47.2	53.5	1.72	1.35	2.20	<0.0001^‡^	1.98	1.40	2.81	0.0001^‡^
	> 65 years	1359	44.6	41.9	47.2	1.53	1.20	1.94	0.0006^‡^	1.97	1.38	2.81	0.0002^‡^
**Sex**	Male^†^	1708	46.1	43.8	48.5								
	Female	876	43.4	40.1	46.7	0.94	0.08	1.03	0.1966	1.05	0.86	1.29	0.6195
**Admission**	Urgent^†^	1183	37.0	34.3	39.8								
	Programmed	1401	52.1	49.5	54.7	1.40	1.28	1.53	<0.0001^‡^	2.58	2.09	3.20	<0.0001^‡^
**Hospitalization length**	< 7 days^†^	1000	27.3	24.5	30.1								
	≥ 7 days	1584	56.5	54.1	58.9	2.07	1.85	2.31	<0.0001^‡^	4.13	3.29	5.18	<0.0001^‡^
**Correctly documented medical records**	Yes^†^	649	35.0	31.3	38.6								
	No	1932	48.7	46.5	50.9	1.39	1.24	1.56	<0.0001^‡^	2.01	1.59	2.55	<0.0001^‡^
**Correctly documented nursing records**	Yes^†^	2474	45.1	43.1	47.0								
	No	107	49.5	40.1	59.0	1.10	0.90	1.34	0.4185	1.21	0.78	1.88	0.3952
**Working day**	Yes^†^	1971	41.5	39.3	43.7								
	No	613	57.1	53.2	61.0	1.38	1.23	1.50	<0.0001^‡^	1.91	1.56	2.34	<0.0001^‡^
**Service**	Angiology and Vascular Surgery	592	51.7	47.6	55.8	1.96	1.54	2.49	<0.0001^‡^	1.25	0.95	1.65	0,1055
	Heart Surgery	221	59.7	53.0	66.4	2.71	1.99	3.70	<0.0001^‡^	1.75	1.25	2.45	0.0010^‡^
	General Surgery 'Service A'	358	64.3	58.3	70.4	1.58	1.21	2.07	0.0008^‡^	1.91	1.44	2.53	<0.0001^‡^
	General Surgery 'Service B'	219	40.6	33.9	47.4	1.25	0.92	1.71	0.1580	1.07	0.76	1.51	0.7101
	Laparoscopic Surgery	103	32.0	22.5	41.5	0.86	0.56	1.32	0.4903	0.78	0.49	1.22	0.2743
	Thoracic Surgery	322	68.9	63.7	74.2	4.06	3.04	5.41	<0.0001^‡^	2.52	1.83	3.49	<0.0001^‡^
	Gynaecology	34	2.9	0.1	15.3	0.06	0.01	0.34	0.0019^‡^	0.10	0.02	0.65	0.0155^‡^
	Neurosurgery	61	36.1	23.2	48.9	1.03	0.62	1.72	0.9072	1.58	0.92	2.71	0.0995
	Ophthalmology	72	31.9	20.5	43.4	0.86	0.53	1.40	0.5404	0.86	0.51	1.43	0.5580
	Otorhinolaryngology	129	51.2	42.1	60.2	1.91	1.32	2.77	0.0005^‡^	0.95	0.64	1.43	0.8214
	Traumatology	252	23.4	18.0	28.8	0.56	0.40	0.78	0.0005^‡^	1.05	0.74	1.49	0.7858
	Urology	221	22.2	16.5	27.9	0.52	0.37	0.74	0.0002^‡^	0.82	0.56	1.20	0.3052

**TOTAL**		2584	45.2	43.3	47.1								

* 95CI: 95% confidence interval; LL: lower limit; UL: upper limit; RR: relative risk; ROR: risk odds ratio. ^† ^reference category. For variable 'Service' the reference is the mean effect. ^‡ ^statistically significant for p < 0.05.

The multivariate analysis maintained the pattern of associations that appeared in the bivariate analysis. Weekends increase the risk of inappropriateness with respect to working days by 91%; programmed admissions showed a risk odds ratio of 2.58 with respect to urgent admissions; the over 65 age group and that of 45 to 65 exhibit inappropriateness rates almost twofold those of the under 45 age group; hospital stays over 7 days showed rates 4.13 times higher than inappropriateness of shorter stays; while incorrect or incomplete medical records had inappropriateness rates double those of correct records. Sex and nursing records showed no association with inappropriateness (table 2).

## Discussion

The figures of inappropriateness for hospital stays are between 10% and 40% in centres with similar characteristics to those contemplated in this analysis [8-12]. The maximum acceptable rate of inappropriateness should be below the rate for the total period of hospitalization which, for the hospital and period under consideration was 34.2% (CI95% 33.3–35.1) [13], even though no standards have been set in this sense. With respect to these data, the rate of inappropriateness shown by our study is high. Furthermore, there are large differences in the number of preoperatory days and the number of them considered to be inappropriate. This would indicate a great variability in the efficiency of care provision, as seen from the point of view of appropriateness.

The evaluation of the factors associated with inappropriate use has scarcely been documented in our setting. In particular, age as a factor associated with inappropriate stays is especially important in our region, where, according to demographic data from 2005 provided by the National Institute of Statistics, 22.6% of the population is over 65 and 3.0% is over 85 years of age, as opposed to nationwide figures of 16.6% and 1.8% respectively. This direct relation between age and the greater rate of inappropriateness had not previously been found in the literature. Although this may be confounded by social factors, it can also serve as a proxy for evaluating the risk at admission.

The association of inappropriateness with weekends is justified by schedule problems, the carrying out of diagnostic tests and the decision making process. At the same time, the association with programmed admission, repeatedly found in various papers  [3,9,14], can be explained by similar reasons. Finally, as expected, long preoperatory stays show an even higher rate of inappropriateness [15].

We have observed that the keeping of correct medical records by the doctor responsible for the patient is associated with a lower rate of inappropriateness. However, it cannot be said that avoiding inappropriate stays is solely due to doctors' professionalism, since the AEP protocol is closely related to the information available in the clinical records, so the aforementioned finding is probably the product of both factors.

The significant associations found between inappropriateness of stay and the admission to services, as for instance 'Heart surgery' or 'Thoracic surgery' or the lower inappropriateness rate found in 'Gynaecology', could be the result of differences in the clinical condition being suffered. However, the association found with admission to just one of the general surgery services reveals the importance of clinical management issues.

To accurately evaluate the effect of other potential confounders in the appropriateness of preoperatory stays, such as bed occupancy, scheduled-unscheduled surgical procedures ratio, psycho-social factors, clinical conditions and comorbididy, would require further investigation. This is not a concurrent review, so inappropriateness may be somewhat overestimated due to the fact that the AEP questionnaire can lead to such an overestimation when the stay is not justified. Variables acting as indicators of the quality of the clinical records could be an approximation for the adjustment. Other limitations derived from the use of the AEP as the measuring instrument are widely described in a number of papers [16-19]. Seasonal variations in rates of appropriateness should not be a limitation of this work as it was carried out using a random sample over a one-year period.

There is a need to establish standards with reference to appropriate use of hospitalization. Those standards should be stricter for periods in which the healthcare provision could be given far more easily at primary care level, as is the preoperatory period, and which, paradoxically, shows higher levels of inappropriateness, mainly with programmed admissions.

## Conclusion

This study leads us to conclude that the inappropriate use of hospital stay during preoperatory care affects almost half the period and that there are some risk determinants that could act as indicators at admission. Further research is needed to evaluate some confounders such as social and clinical factors.

## Competing interests

The author(s) declare that they have no competing interests.

## Authors' contributions

ST, APR, JJC, MBCA, FJL and SSS conceived the study and participated in its design and coordination, collected the data, performed the statistical analysis and helped to draft the manuscript. PLE, MPTP and JMGG participated in the study coordination and helped to draft the manuscript. All authors read and approved the final manuscript.

## Pre-publication history

The pre-publication history for this paper can be accessed here:


